# NRLC-YOLO for lightweight detection and grasp positioning of latex cups in rubber plantations

**DOI:** 10.3389/fpls.2026.1836301

**Published:** 2026-05-08

**Authors:** Ruiwu Xu, Xirui Zhang, Yulan Liao, Zhifu Zhang, Junxiao Liu

**Affiliations:** 1 School of Information and Communication Engineering, Hainan University, Haikou, Hainan, China; 2 School of Mechanical and Electrical Engineering, Hainan University, Haikou, Hainan, China

**Keywords:** Hevea brasiliensis, intelligent agriculture, lightweight deep learning, object detection, robotic harvesting

## Abstract

Natural rubber harvesting remains highly dependent on manual labor, particularly during latex cup collection, which limits efficiency and increases operational costs. Intelligent robotic harvesting systems require accurate visual perception and reliable grasp point positioning under rubber plantation environments. However, latex cups are typically small, visually diverse, and often affected by adjacent latex drains, making detection and manipulation challenging for existing vision models. To address these challenges, this study proposed a lightweight vision-based framework for latex cup detection and grasp-point positioning in automated rubber harvesting. The proposed NRLC-YOLO is developed based on YOLO11n-seg by integrating a lightweight backbone, a local–global attention enhancement module, and a dynamic convolution strategy. In addition, a fast grasp-point positioning method is designed to determine the gripping center of latex cups, enabling stable robotic manipulation. As validated by experimental results, our model yields a mAP*_@50–95_* of 78.5%, which surpasses the baseline approach with fewer parameters and lower computational costs. The average error of the proposed grasp-point positioning scheme is measured at 8.08 pixels. Field experiments further demonstrate a grasping success rate of up to 93.3% under real plantation conditions. The proposed framework provides an efficient AI-enabled perception solution for automated latex cup harvesting and offers practical support for the development of intelligent rubber plantation management systems.

## 1 Introduction

Natural rubber, extracted from Hevea brasiliensis (Ali et al., 2020), is an essential industrial raw material (Kunam et al., 2023) widely used in transportation, medical products, and advanced manufacturing due to its elasticity, durability, and biocompatibility (Bi et al., 2022; de Lima et al., 2023; Pamornnak et al., 2020). However, rubber production remains highly dependent on manual operations, especially during latex cup installation and collection (Wang et al., 2022). This labor-intensive harvesting mode not only limits operational efficiency but also poses increasing challenges in regions facing labor shortages and rising labor costs (Yang et al., 2022). Consequently, enhancing latex harvesting efficiency, decreasing reliance on manual labor, and developing an automated system for collecting latex cups have become urgent priorities.

**Figure 1:**
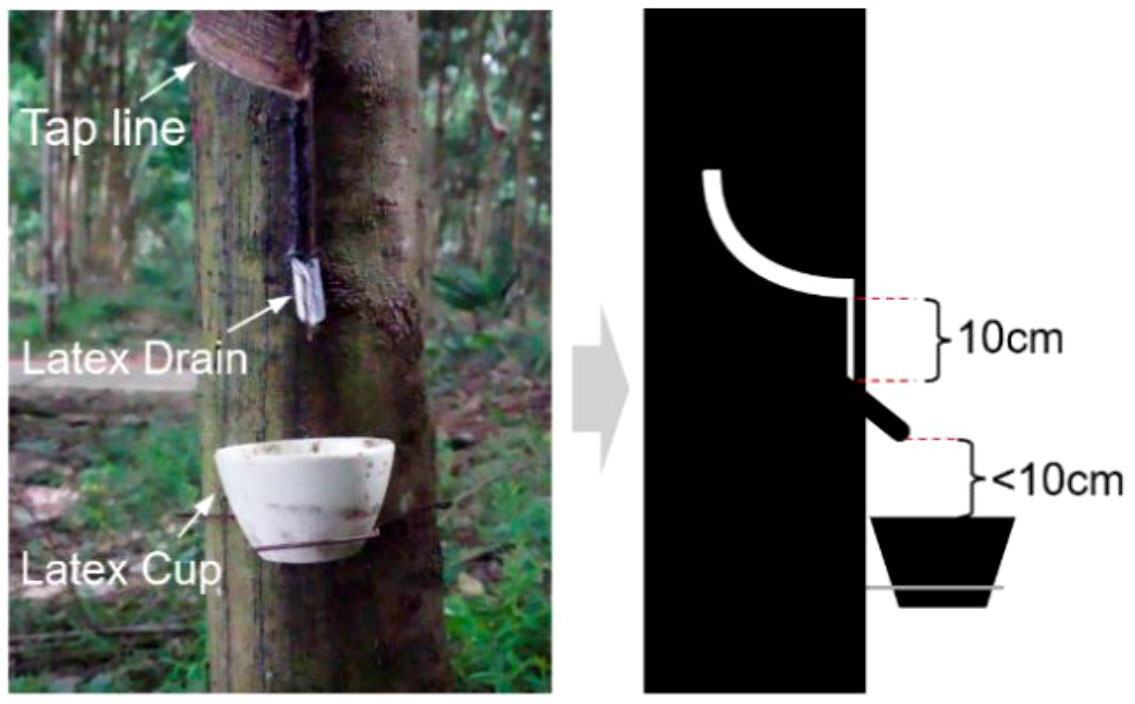
Agronomic requirements of the latex cup.

In automated latex harvesting, accurate detection and positioning of latex cups represent a critical prerequisite for reliable robotic grasping. According to agronomic requirements (Sheil et al., 2025), latex cups are typically installed several centimeters below the tapping cut to prevent latex splashing and contamination (**Figure 1**).

However, in real plantation environments, latex cups are often affected by the presence of latex drains, while variations in cup color and size further increase visual complexity. These characteristics make latex cups a typical small-scale agricultural target with complex background interference, posing significant challenges for visual perception systems (Zhang et al., 2025a). Inaccurate positioning may result in grasping failure, latex contamination of mechanical components, and reduced harvesting efficiency, thereby undermining the practicality of automated harvesting systems (Hua et al., 2025).

Existing studies have explored deep learning-based object detection and segmentation methods for agricultural perception tasks (Dong et al., 2025; He et al., 2025; Yang et al., 2024). YOLO-based lightweight frameworks have been widely adopted due to their efficiency and real-time performance (Li et al., 2025a; Liu et al., 2024). Nevertheless, directly applying existing detection models to latex cup harvesting remains challenging. On the one hand, the small size and irregular appearance of latex cups significantly degrade detection performance (Zhang et al., 2025b). On the other hand, lightweight backbone replacement often leads to the loss of fine-grained features, which is critical for accurately identifying small-scale objects (Yang et al., 2025; Zhang et al., 2025c). Consequently, how to balance detection accuracy and lightweight deployment is still a key unresolved issue in the field of agricultural vision.

Latex cup harvesting differs from generic object detection tasks in that it requires precise localization of the graspable region to ensure stable gripping without damaging the cup or contaminating the latex. In this context, traditional computer vision techniques still have practical value, especially for lightweight geometric localization after grasp-related regions have been extracted (Jia et al., 2024; Yuan et al., 2025). Methods based on handcrafted features and geometric constraints are computationally efficient, interpretable, and easy to integrate into real-time robotic control (Lin et al., 2025). However, when used alone for target perception in plantation environments, their performance is often limited by illumination variation, background clutter, and object appearance diversity (Li et al., 2024).

Meanwhile, deep learning-based end-to-end grasping methods have demonstrated promising performance in robotic manipulation (Ci et al., 2024). However, their direct application to latex cup harvesting remains challenging due to the need for large-scale grasp annotations and their limited interpretability and generalization in rubber plantation environments.

To tackle the above challenges, this study presents a lightweight and high-precision framework for latex cup detection and grasp-point localization. The primary contributions of this work are summarized as follows:

1. A lightweight instance-segmentation framework is developed for latex cup harvesting in rubber plantations, aiming at the dual challenge of small-target perception and real-time robotic deployment.
2. A representation-compensation strategy is designed to alleviate the feature degradation caused by lightweight backbone replacement, by combining local–global attention enhancement and omni-dimensional dynamic convolution, thereby improving robustness under rubber plantation backgrounds.
3. A fast mask-driven grasp center positioning method is proposed to convert segmentation outputs into graspable 3D positions without iterative optimization, enabling efficient robotic harvesting in field conditions.

## 2 Materials and methods

### 2.1 Dataset construction and annotation

**Figure 2:**
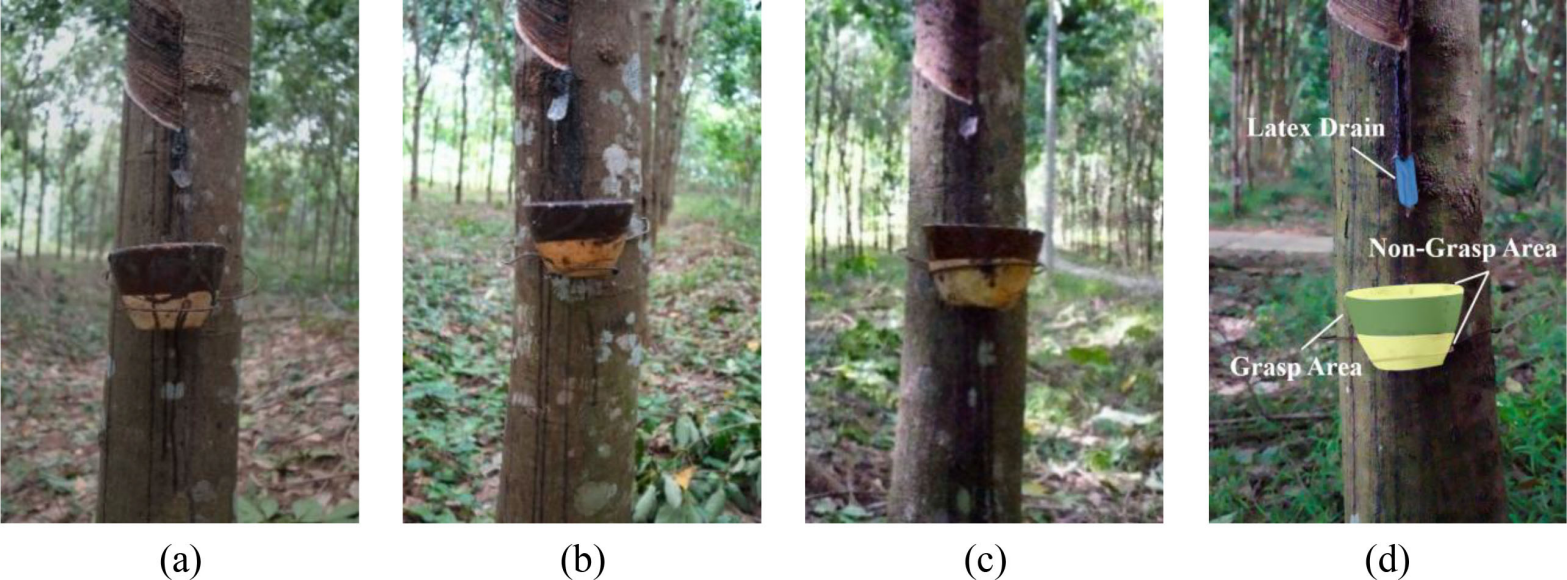
Natural rubber latex cup dataset and annotation: **(a)** 7:00 to 8:00 AM images; **(b)** 8:00 to 9:00 AM images; **(c)** 9:00 AM to 10:00 AM images; **(d)** detection area annotation.

The dataset used in this study was collected at the Natural Rubber Experimental Base of Hainan University (19°30′N, 109°29′E). Images were captured using a Sony Alpha 7III camera with a resolution of 3000 × 4000 pixels. To ensure representativeness under real harvesting conditions, data acquisition was conducted across three different plantation areas and under varying natural illumination conditions. A total of 2000 images were acquired during the period from 7:00 to 10:00 in the morning, as shown in **Figures 2a-c**.

The Labelme annotation tool was used to annotate the grasped and non-grabbed areas of the latex drain and latex cup, as shown in **Figure 2d**. The dataset was randomly partitioned into training and validation subsets with a split ratio of 7:3. To improve the generalization ability of the model under the current dataset scale, the default data augmentation strategy of the YOLO framework was adopted during training, including random scaling, horizontal flipping, and color-space perturbation, which enhanced the robustness of the model to illumination variation, scale change, and background interference in rubber plantation environments. The detailed annotation statistics are presented in **Table 1**.

**Table 1:** Dataset description.

Dataset	Images	Instances		
		Latex drain	Grasp area	Non-Grasp area
Train	1400	1260	1400	2673
Test	600	540	600	1281
Total	2000	1800	2000	3954

### 2.2 NRLC-YOLO model structure

**Figure 3:**
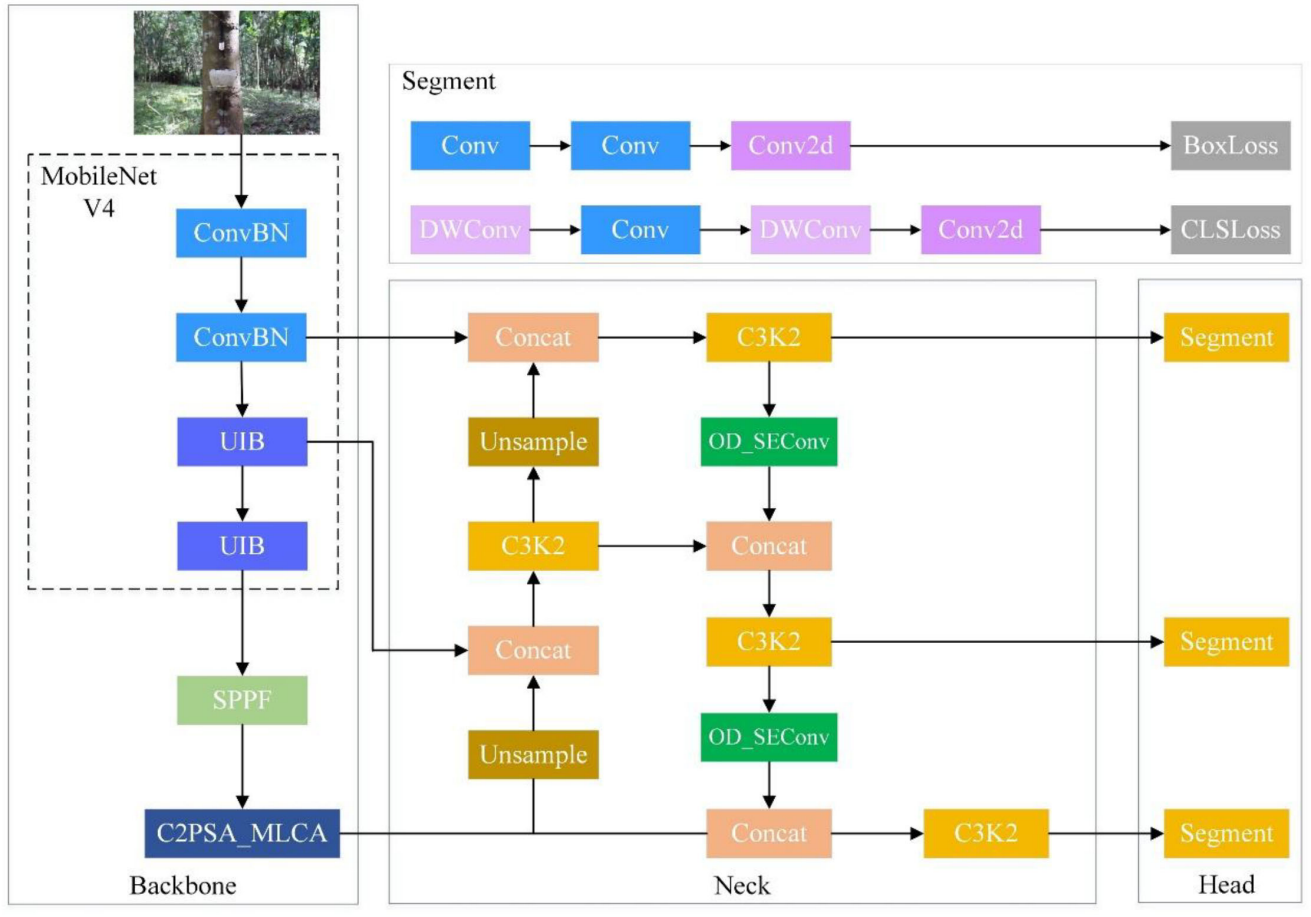
NRLC-YOLO model structure.

To achieve accurate and efficient detection of grasp-related regions of latex cups in rubber plantation environments, an improved lightweight instance segmentation model, termed NRLC-YOLO, was developed based on YOLO11n-seg, as shown in **Figure 3**.

Different from the original YOLO11n-seg, which is designed for general-purpose instance segmentation, NRLC-YOLO is a task-specific redesign for latex cup harvesting. The difference lies not only in module replacement, but also in the adaptation of the feature extraction framework to the practical requirements of this task. In rubber plantations, latex cups usually present small target size, appearance variation, and interference from latex drains and surrounding backgrounds. Meanwhile, the model must satisfy the real-time and lightweight deployment requirements of robotic systems. Therefore, the proposed NRLC-YOLO is designed to achieve a balance between computational efficiency, small-target perception, and robustness in complex scenes.

Specifically, MobileNetV4 is adopted as the backbone to reduce model complexity. To alleviate the feature representation loss caused by backbone lightweighting, the C2PSA_MLCA module is introduced to enhance both local detail perception and global contextual modeling. In addition, OD_SEConv is incorporated to improve adaptive feature extraction under complex backgrounds. Through these modifications, NRLC-YOLO is better suited than YOLO11-seg for real-time latex cup detection and grasp-related region segmentation in robotic harvesting applications.

#### 2.2.1 MobileNetV4

**Figure 4:**
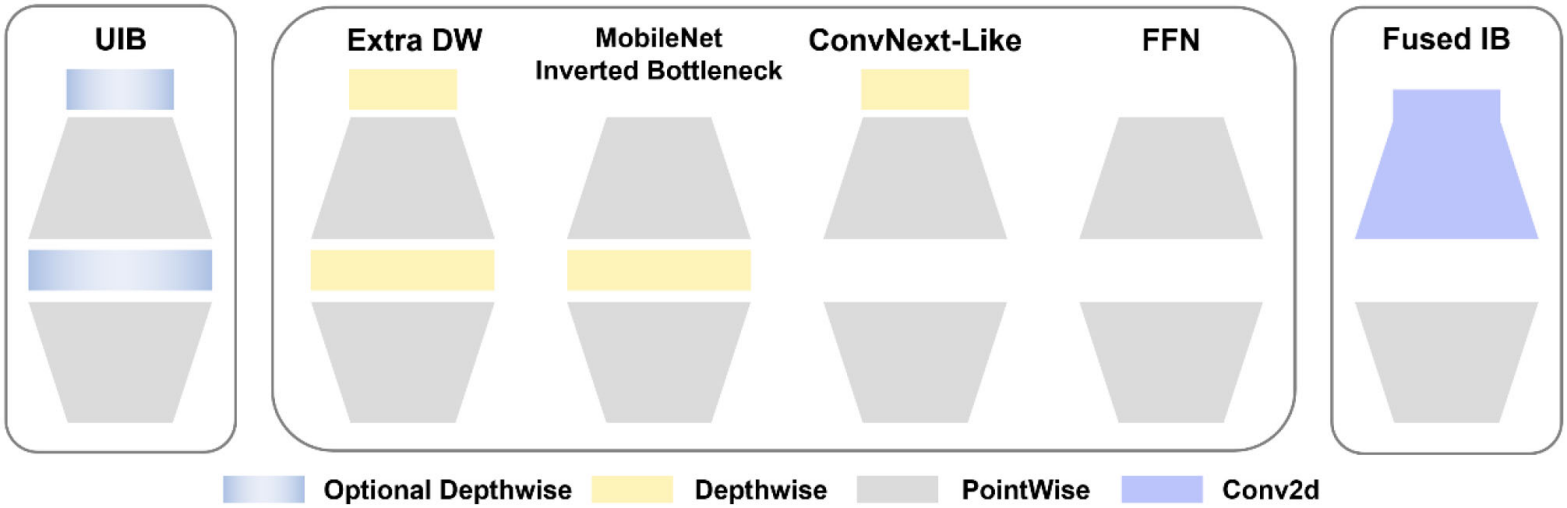
Universal Inverse Bottleneck (UIB) search module.

MobileNetV4 (Qin et al., 2024) introduces the Universal Inverse Bottleneck (UIB) (Chen et al., 2026) search module, which integrates the inverted bottleneck, ConvNext, feedforward neural network, and innovative extra depth (ExtraDw). The schematic diagram of the UIB Block structure is shown in **Figure 4**. Another innovation is the Mobile MQA attention mechanism. Mobile MQA significantly improves the inference speed on mobile accelerators by optimizing the ratio of arithmetic operations to memory accesses. The mathematical definition of Mobile MQA is presented below:

(1) $$MobileMQA(x)=Concat(a_{1}\ldots a_{n})W^{O}$$

(2) $$attention_{j}=(\frac{(XW^{Q_{j}}){\left(SR(X)W^{k}\right)}^{T}}{\sqrt{d_{k}}}){\left(SR(X)W^{k}\right)}^{V}$$

here SR stands for spatial reduction, which involves asymmetric spatial downsampling of keys and values to further improve efficiency.

#### 2.2.2 C2PSA_MLCA

**Figure 5:**
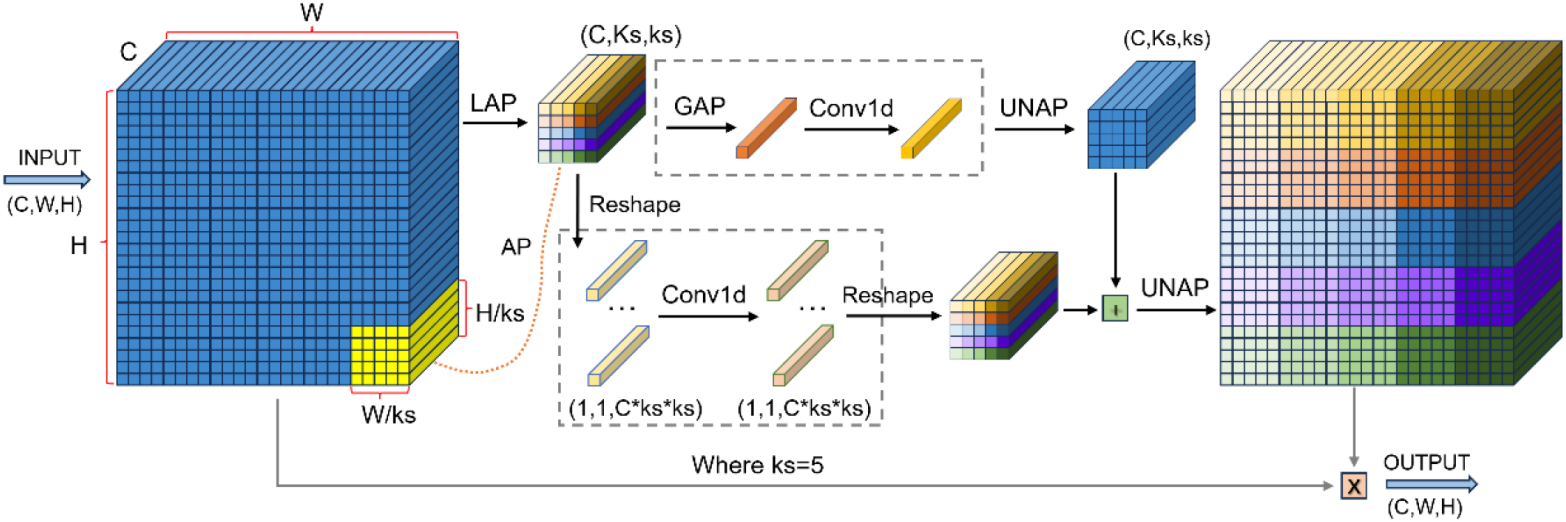
MLCA attention mechanism process.

To address the degradation in detection accuracy caused by model lightweighting when detecting latex cups, this study introduces a Mixed Local Channel Attention (MLCA) mechanism (Wan et al., 2023) into the YOLO11-seg architecture. MLCA is capable of effectively integrating fine-grained local spatial information with global contextual cues while maintaining low computational overhead, thereby enhancing the network’s ability to discriminate key features of small and irregular agricultural targets. The overall structure of the MLCA module as shown in **Figure 5**.

The fundamental mechanism is built upon a multi-head attention framework, which first focuses on local spatial details and then highlights salient channel-wise features. In this way, local and global information is effectively fused to improve both feature representation capability and extraction efficiency. The formulation was given as follows:

(3) $$k=\phi (C)=|\frac{\log_{2}(C)}{\gamma }+\frac{\text{b}}{\gamma }{|}_{odd}$$

here, k represents the kernel size of the 1D convolution, C stands for the number of channels, γ, b are hyperparameters both set to 2 by default. Following the original work by using the default value of 2 helps effectively highlight salient regions while preserving global contextual information.

(4) Output=UNAP(Convld(LAP(X))+)UNAP(Convld(GAP(X)))

here *X* denotes the input feature vector, LAP represents local average pooling, signifies global average pooling, Convld indicates 1D convolution, UNAP refers to Unaverage pooling.

**Figure 6:**
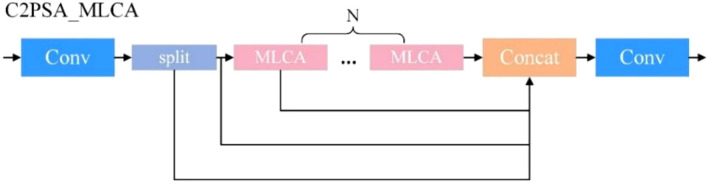
C2PSA_MLCA module.

In this study, the original attention layer in the C2PSA module of the YOLO11-seg model is replaced with the MLCA mechanism, forming the C2PSA_MLCA module. By jointly exploiting local spatial cues and global contextual information in both channel and spatial dimensions, the proposed C2PSA_MLCA module effectively compensates for the representational capacity loss introduced by backbone lightweighting, thereby improving detection accuracy for latex cups without significantly increasing computational complexity. The detailed network structure of the C2PSA_MLCA module is shown in **Figure 6**.

#### 2.2.3 OD_SEConv

**Figure 7:**
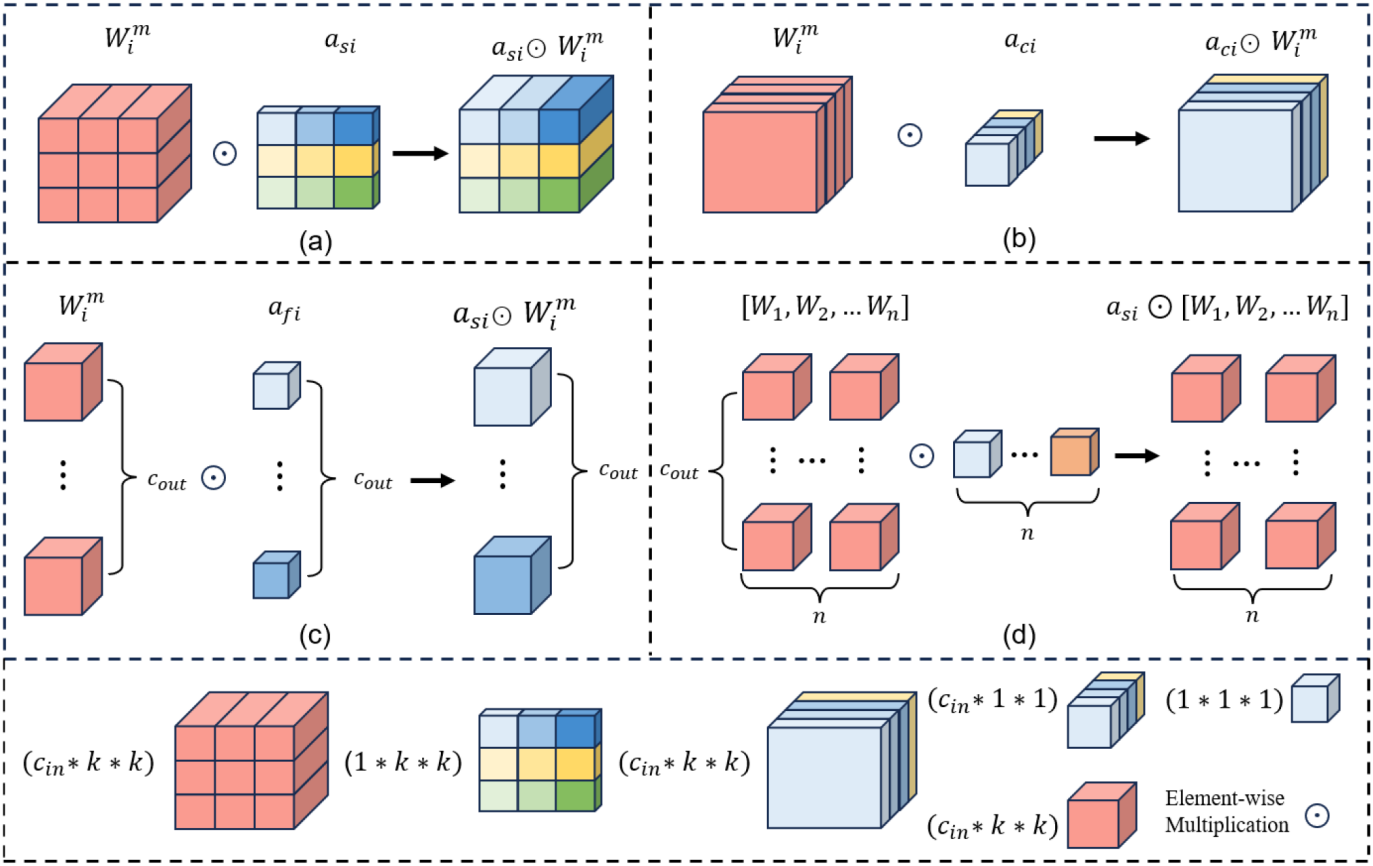
OD_Conv multi-dimensional attention mechanism.

To improve small-target feature extraction for latex cups while maintaining the lightweight property of the model, this study introduces omni-dimensional dynamic convolution (Li et al., 2022) (OD_Conv). Its structure is shown in **Figure 7**.

Different from traditional convolution, which applies fixed kernels to all input samples, OD_Conv performs input-adaptive convolutional modulation by learning attention weights from the current feature map. In this way, the convolution operation can dynamically adjust to different input characteristics, thereby improving the flexibility of feature extraction. This property is particularly beneficial for latex cup detection, since latex cups usually exhibit small target size, appearance variation, partial occlusion, and interference from surrounding latex drains and plantation backgrounds.

Compared with traditional convolution operations, OD_Conv is capable of dynamically generating four-dimensional weight parameters, which enables it to more effectively address the issues of inflexible feature extraction and the loss of fine-grained details in small targets. The specific calculation procedure is described as follows:

First, the input feature map $x\in {\mathbb{R}}^{C_{in}\times H\times W}$is compressed in terms of spatial information through global average pooling, yielding a channel descriptor vector $z\in {\mathbb{R}}^{C_{in}}$:

(5) $$z_{c}=\frac{1}{H\cdot W}\sum_{i=1}^{H}\sum_{j=1}^{W}x_{c}(i,j)$$

vector z is then dimensionality-reduced through a shared fully connected (FC) layer with reduction ratio r=1/16, followed by a ReLU activation function, yielding transformed vector $z_{t}$:

(6) $$z_{t}=\delta (W^{\left(1\right)}\cdot z)$$

Where $W^{\left(1\right)}\in {\mathbb{R}}^{d\times C_{in}}$is the shared FC weight matrix, $d=C_{in}/r$ denotes the reduced dimensionality (r=16), δ(·) represents the ReLU activation.

Subsequently, $z_{t}\in {\mathbb{R}}^{C_{in}/r}$is processed by four parallel attention branches. Each branch contains an FC layer and activation function, respectively producing:

(7) $$\left\{ \begin{array}{l} \alpha_{si}=Sigmoid(W_{s}^{\left(2\right)}z_{t}),W_{s}^{\left(2\right)}\in {\mathbb{R}}^{K_{h}\cdot K_{w}\cdot d}\\ \alpha_{si}=Sigmoid(W_{s}^{\left(2\right)}z_{t}),W_{s}^{\left(2\right)}\in {\mathbb{R}}^{K_{h}\cdot K_{w}\cdot d}\\ \alpha_{si}=Sigmoid(W_{s}^{\left(2\right)}z_{t}),W_{s}^{\left(2\right)}\in {\mathbb{R}}^{K_{h}\cdot K_{w}\cdot d}\\ \alpha_{si}=Sigmoid(W_{s}^{\left(2\right)}z_{t}),W_{s}^{\left(2\right)}\in {\mathbb{R}}^{K_{h}\cdot K_{w}\cdot d} \end{array}\right.$$

Finally, OD_Conv is formulated as Equation 8, where $\alpha_{si},\alpha_{ci},\alpha_{fi},$ and $\alpha_{wi}$ hierarchically multiply the convolutional kernel $W_{i}$. This enables differentiation across all four input dimensions, achieving multidimensional dynamic adaptation.

(8) $$y=(\alpha_{s1}⊙\alpha_{c1}⊙\alpha_{f1}⊙\alpha_{w1}⊙W_{1}+\ldots +\alpha_{sn}⊙\alpha_{cn}⊙\alpha_{fn}⊙\alpha_{wn}⊙W_{n})*x$$

However, in the original OD_Conv, the input channel attention is generated by a relatively lightweight linear transformation, which is insufficient for modeling complex inter-channel dependencies under plantation environments. In addition, after backbone lightweighting, the discrimination of fine-grained target features may be further weakened, making small latex cup regions more susceptible to background interference.

**Figure 8:**
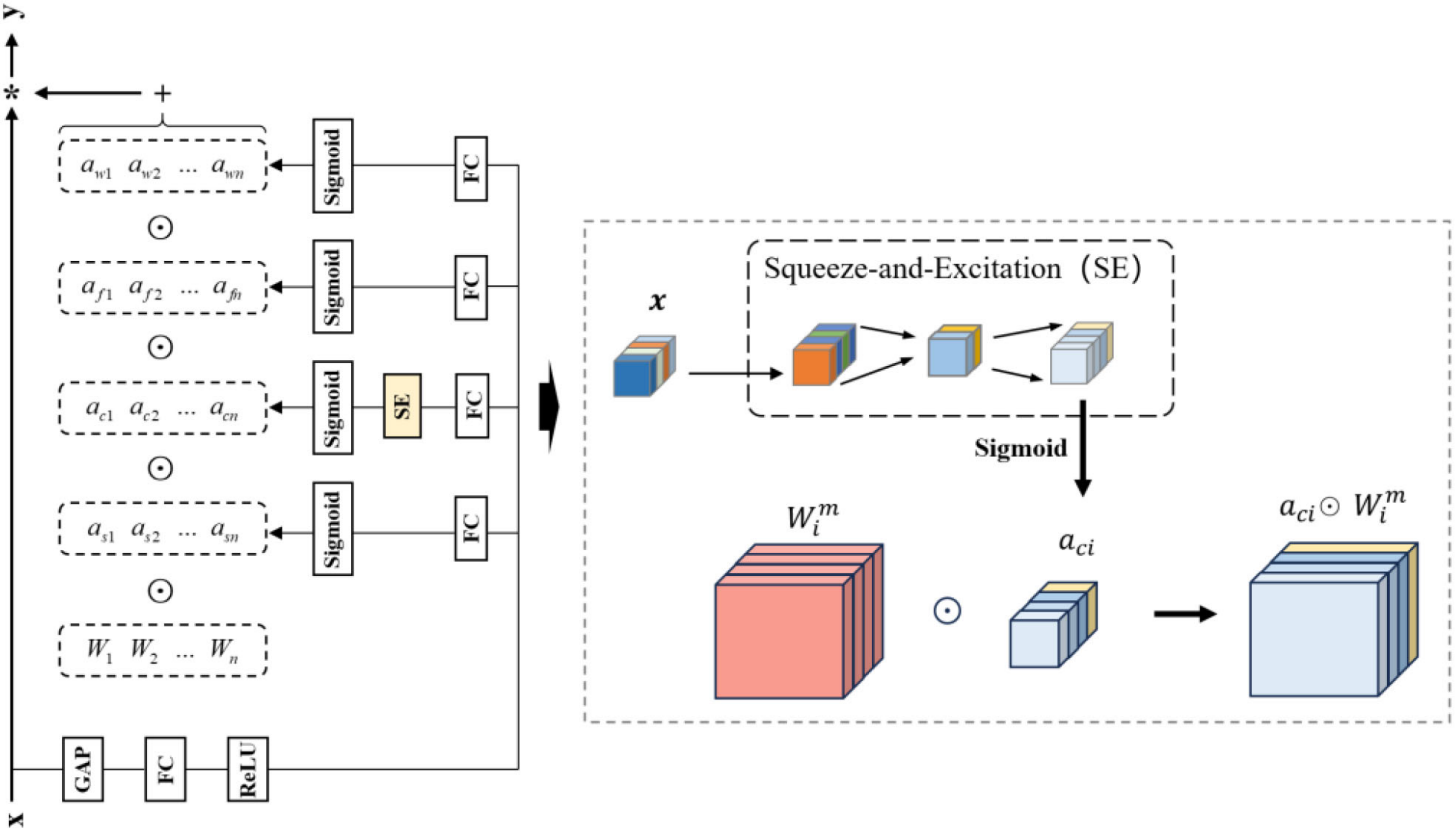
OD_SEConv structure.

To address this limitation, an OD_SEConv module is proposed, as shown in **Figure 8**. Specifically, a lightweight squeeze-and-excitation (SE) branch is introduced before OD_Conv to explicitly model global channel dependencies and recalibrate the channel responses of the input feature map. Since the input and output dimensions of the SE branch remain consistent with those of OD_Conv, it can be embedded without changing the feature size. By performing channel reweighting before dynamic convolution, informative channels related to latex cup structures can be enhanced, while irrelevant background responses can be suppressed.

Therefore, the proposed OD_SEConv can be regarded as a two-stage adaptive refinement process. The SE branch first strengthens global channel dependency modeling, and the subsequent OD_Conv further performs input-adaptive convolutional modulation. This design improves the representation of small and visually variable latex cup targets, enhances robustness to complex plantation backgrounds, and introduces only limited additional computational cost.

#### 2.2.4 Grasp center point positioning method

**Figure 9:**
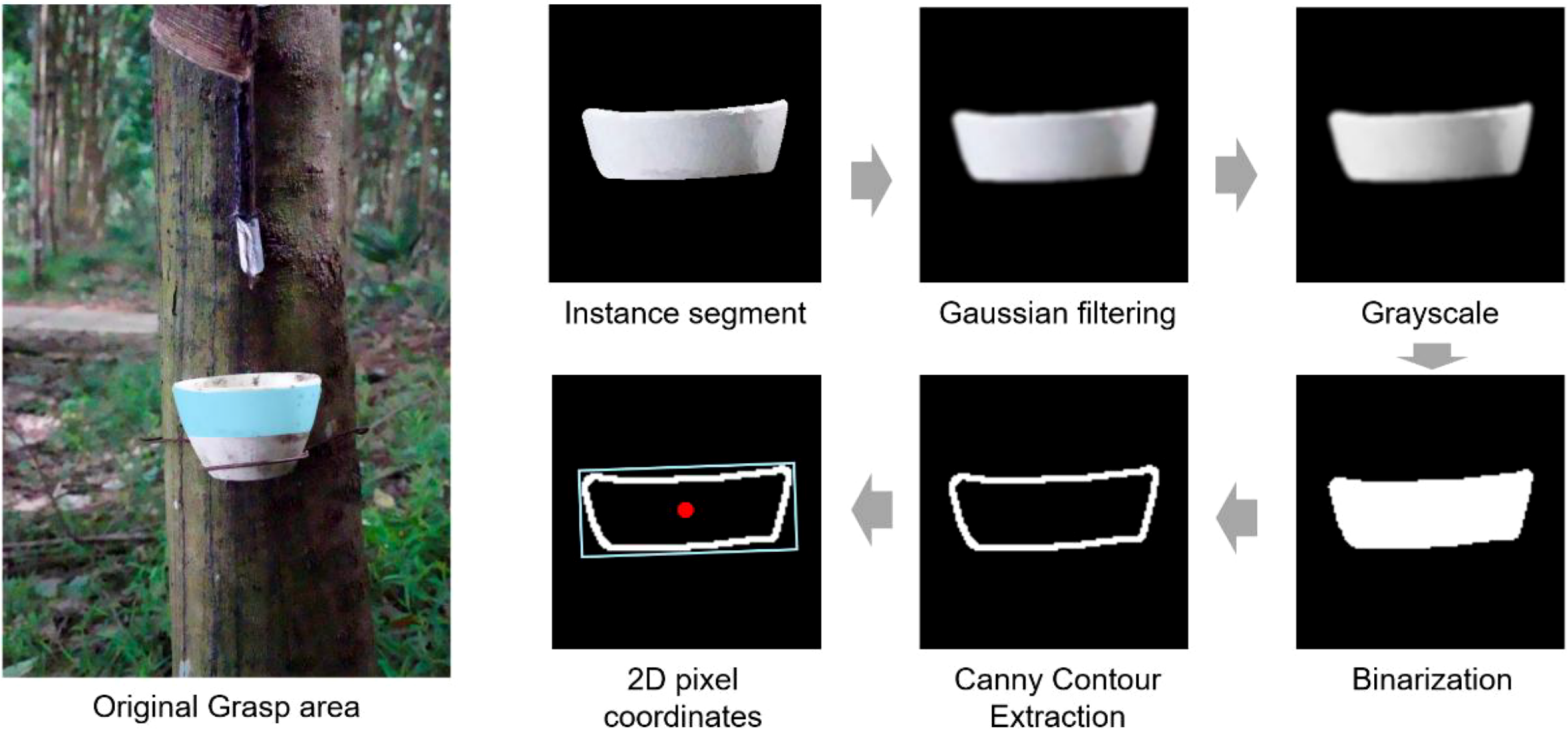
Latex cup center point positioning method.

For the latex cup grasping task, we present a novel center point positioning scheme that relies on the detection output of the NRLC-YOLO model. The detailed pipeline is depicted in **Figure 9**.

This method first performs grayscale processing on the segmented image after Gaussian filtering, and then binarizes the grayscale image. The image edge detection and extraction of the binarized image are performed using the Canny operator, the area occupied by the contour is calculated, and the minimum enclosing rectangle is calculated using the cv2.minAreaRect() function in the OpenCV library. By taking the point set of the segmented latex cup grasping area as input, a rotated rectangle is returned, and then converted into the four fixed point coordinates $\left(x_{1},y_{1}\right)$, $\left(x_{2},y_{2}\right)$, $\left(x_{3},y_{3}\right)$, $\left(x_{4},y_{4}\right)$ containing the minimum enclosing rectangle through the cv2.boxPoints() function. The center point $o_{i}(x_{i},y_{i})$ of the i−th minimum bounding rectangle can be obtained by calculating the average coordinates of its four vertices. After obtaining the two-dimensional pixel coordinates, the depth coordinates are measured using the depth camera D455 to finally determine the 3D coordinates $o_{i}(x_{i},y_{i},z_{i})$ of the grasp center point.

Unlike skeleton-based or optimization-driven grasp-point localization methods, the proposed strategy avoids iterative computation and complex geometric modeling, thereby ensuring robustness and computational efficiency for real-time robotic grasping in plantation environments.

## 3 Experiments and results

### 3.1 Experimental setup

The experimental environment employed in this work consists of two components. The first refers to the model validation experiment, which is conducted on a hardware platform equipped with an Intel i7-14700F CPU, 16 GB of RAM, and an NVIDIA RTX 4060Ti GPU. The implementation is based on the PyTorch 2.0.1 framework with Python 3.8.20, and CUDA 11.8 is utilized for GPU acceleration. During model training, all input images are resized to a resolution of 640×640 pixels. The training process is executed for up to 300 epochs, with an early stopping strategy applied: training terminates automatically if no performance improvement is observed within 100 consecutive epochs, and the corresponding model is regarded as the optimal one. Additional training hyperparameters and the default YOLO data augmentation settings are summarized in **Table 2**.

**Table 2:** Training hyperparameters and default data augmentation settings.

Hyperparameter	Value	Hyperparameter	Value
batch size	16	box	7.5
lr0	0.01	cls	0.5
lrf	0.01	dfl	1.5
momentum	0.937	fliplr	0.55
weight_decay	0.0005	scale	0.5
hsv_h	0.015	hsv_s	0.7
hsv_v	0.4	augmentation	default YOLO

**Figure 10:**
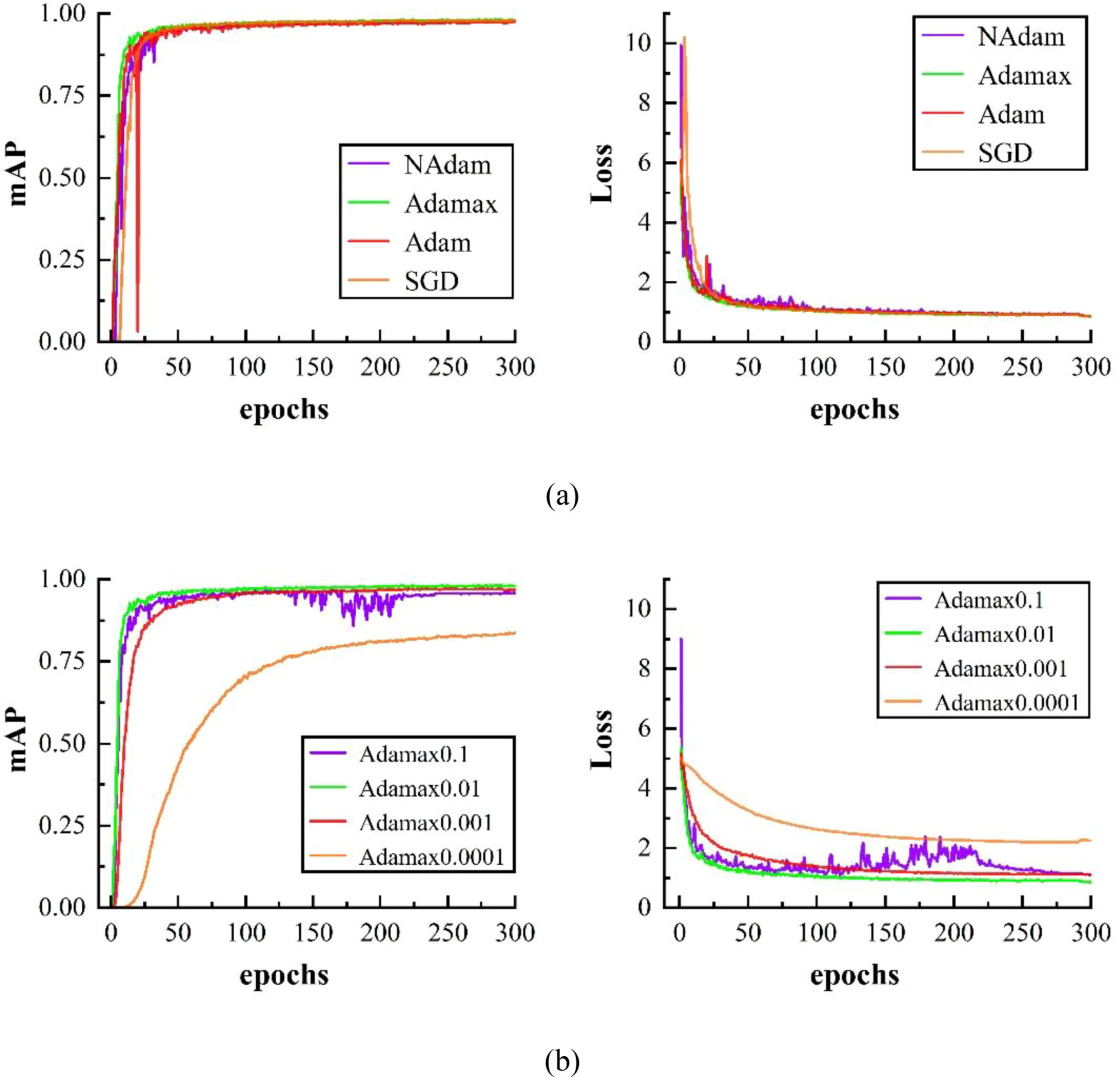
Performance investigation under different optimizers and initial learning rates: **(a)** Results obtained by four optimizers at the learning rate of 0.01; **(b)** Performance comparison under distinct initial learning rates based on the Adamax optimizer.

The learning rate regulates the update amplitude of network parameters during each iteration. The optimizer is adopted to iteratively refine model parameters toward a smaller loss value. As depicted in **Figure 10a**, an initial learning rate of 0.01 was employed, and four optimizers (SGD, Adam, Adamax, NAdam) were evaluated. Experimental results indicate that the Adamax optimizer delivers superior detection performance and smaller loss, with convergence speed similar to other schemes. Thus, Adamax was adopted as the optimizer in this work.

**Figure 10b** illustrates the performance of Adamax under various learning rates. A value of 0.0001 results in degraded detection precision and larger loss, while 0.1 leads to unstable convergence and severe fluctuations. It is verified that 0.01 achieves the optimal overall effect. After sufficient comparison experiments, the Adamax optimizer with an initial learning rate of 0.01 was determined as the final training setting.

**Figure 11:**
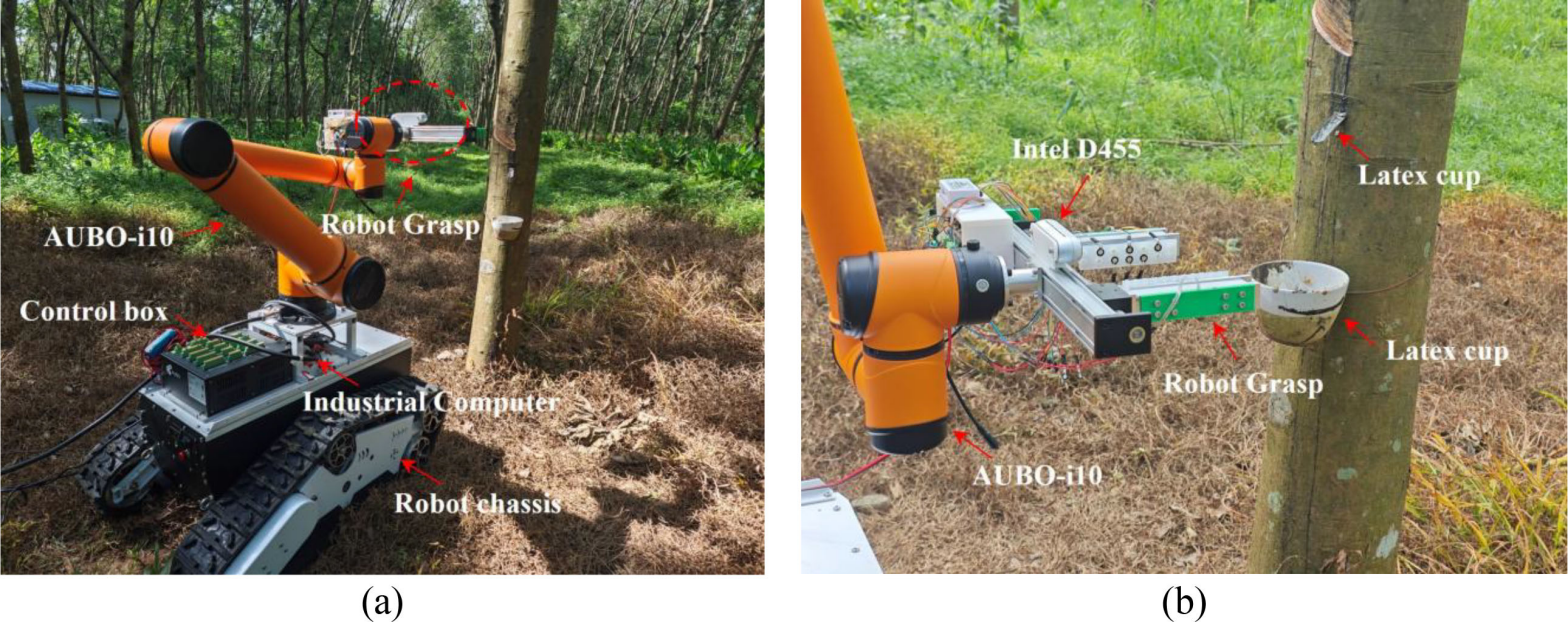
Forest experiment configuration: **(a)** Robot forestry operations; **(b)** End effector configuration.

In the forest grasping verification experiment, the experimental equipment consisted of a robot chassis, an AUBO-i10 robotic arm, a robot gripper, an Intel D455 camera, etc., and the specific configuration is shown in **Figure 11**. Meanwhile, before conducting the forest experiment, the ROS easy_handeye package was used to perform standard hand-eye calibration using a checkerboard pattern to establish the transformation relationship between the camera coordinate system and the robot end effector coordinate system.

### 3.2 Model evaluation metrics

To systematically and objectively assess the overall performance of the NRLC-YOLO model, we conducted validation experiments on both the latex cup key-point detection module and the grasp-center positioning approach. Precision, Recall, Params, and mAP were adopted as the main evaluation metrics for key-point detection (Li et al., 2025b). Meanwhile, GFLOPs and Params were utilized to quantify the computational complexity and model size. The corresponding mathematical definitions of these evaluation metrics are given in Equations 9-13.

(9) $$P=\frac{TP}{TP+FP}$$

(10) $$R=\frac{TP}{TP+FN}$$

(11) $$mAP=\frac{1}{N}\sum_{i=1}^{N}AP_{i}$$

(12) $$FLOPs=2\times C_{in}\times C_{out}\times K_{w}\times K_{h}\times H_{out}\times W_{out}$$

(13) $$Params=(K_{h}\times K_{w}\times C_{in})\times C_{out}$$

here *TP* denotes the number of latex cup key regions correctly identified by the model, *FP* denotes the number of background regions incorrectly identified as target regions, and *FN* denotes the number of ground-truth key regions missed by the model. $K_{h}$ *K_w_* are the convolution kernel sizes, $C_{in}$, $C_{out}\;$are the number of input and output channels, and $H_{in},H_{out}$ are the output feature map sizes.

The quality of grasp localization can be measured by computing the Euclidean distance between the estimated position and the real target point. The coordinates of the predicted grasping point include the center point of the grasping bounding box $\left(x_{center},y_{center}\right)$. Assuming that the coordinates of the actual grasping point are (x,y) and the image resolution is *W*×*H*, the pixel spacing d is between the two. According to Equation 14, the distance between the two points can be calculated.

(14) $$d=\sqrt{{\left[W\times (x_{center}-x)\right]}^{2}+{\left[H\times (y_{center}-y)\right]}^{2}}$$

The positioning error reported in this study was measured in the resized image space used for model inference and evaluation (640 × 640), rather than directly on the original 3000 × 4000 camera images. All models were evaluated under the same resolution setting to ensure fairness and consistency. For the positioning experiment, a priority matching strategy was employed to establish accurate correspondence between predicted and ground-truth grasp centers, where each ground-truth grasp center was matched to the predicted point with the minimum Euclidean pixel distance. To assess localization validity, a maximum distance threshold of 50 pixels was defined, and only predictions within this threshold were regarded as successful localization results, whereas those exceeding it were considered failures. At the representative operating distance used in the robotic experiments, the 50-pixel threshold approximately corresponds to a physical deviation of about 15 mm. This value is provided only as a practical reference, since the actual physical scale represented by one pixel varies with camera-to-target distance, viewpoint, and depth geometry in field conditions. Unrecognized ground-truth targets were counted as missed detections, while background regions incorrectly identified as objects were treated as false positives.

#### 3.2.1 Ablation experiment

To verify the effectiveness of each improved module, we conducted ablation experiments. Here, the original YOLO11-seg backbone network was replaced with the MobileNetV4 module, the C2PSA module was replaced with C2PSA_MLCA, and the lightweight OD_SEConv was adopted. These three improvement methods were incorporated into the original network model either individually or in combination.

Experimental results are summarized in **Table 3**. As can be observed, replacing the original backbone with MobileNetV4 markedly improves the computational efficiency of the model. The number of parameters is reduced from 6.0 M to 4.1 M, GFLOPs decrease from 10.2 G to 7.8 G, and the inference speed increases from 57.9 FPS to 72.8 FPS, demonstrating the clear advantage of the lightweight backbone in reducing model complexity and improving deployment efficiency. However, this gain in efficiency is accompanied by a decline in detection performance, with precision decreasing from 97.7% to 97.5%, recall from 96.9% to 95.7%, mAP_@50_ from 98.0% to 97.5%, and mAP_@50–95_ from 78.2% to 75.3%. These results indicate that although MobileNetV4 provides a solid foundation for real-time detection, backbone lightweighting also weakens the feature representation capability of the network, especially for fine-grained positioning of small and complex targets.

**Table 3:** Results of ablation experiments.

Mobile_NetV4	C2PSA_MLCA	OD_SEConv	*P* (%)	*R* (%)	*mAP_@50_* (%)	*mAP_@50-95_* (%)	*Params* (M)	*GFLOPs* (G)	*FPS* (f/s)
×	×	×	97.7	96.9	98.0	78.2	6.0	10.2	57.9
✓	×	×	97.5	95.7	97.5	75.3	4.1	7.8	72.8
×	✓	×	97.8	96.5	98.1	77.0	5.9	10.1	58.5
×	×	✓	97.6	96.7	98.0	78.0	6.1	10.3	68.0
×	✓	✓	97.8	96.9	98.2	78.5	6.2	10.4	70.5
✓	×	✓	97.5	96.0	97.9	76.5	4.3	7.9	115.0
✓	✓	×	97.6	96.4	98.1	77.4	4.2	7.9	73.0
✓	✓	✓	97.6	96.8	98.3	78.5	4.4	7.9	126.7

When the functional enhancement modules are introduced individually, C2PSA_MLCA and OD_SEConv exhibit different contribution patterns. Specifically, C2PSA_MLCA slightly improves precision and mAP*_@50_*, indicating that the module enhances discriminative representation by jointly exploiting local spatial details and global channel dependencies. Nevertheless, its standalone effect on recall and mAP*_@50–95_* remains limited, suggesting that its contribution is more reflected in feature selection and salient information enhancement than in direct localization refinement. In contrast, OD_SEConv keeps the overall detection accuracy close to that of the baseline while increasing the inference speed to 68.0 FPS, showing that its main contribution lies in strengthening the adaptive feature extraction capability of convolutional operations with only marginal additional complexity. Therefore, C2PSA_MLCA mainly improves feature discrimination, whereas OD_SEConv mainly enhances convolutional adaptability and feature response efficiency.

The combined ablation results further demonstrate the complementarity of the proposed modules. When C2PSA_MLCA and OD_SEConv are jointly introduced on the original backbone, both detection accuracy and inference speed are improved, confirming that the two modules are mutually beneficial in enhancing feature selection and adaptive convolutional response. Under the lightweight backbone, both MobileNetV4 + C2PSA_MLCA and MobileNetV4 + OD_SEConv can partially recover the performance loss caused by backbone simplification, among which OD_SEConv shows a stronger recovery effect on recall and mAP_@50–95_, indicating that dynamic convolution is more effective in compensating for the loss of fine-grained feature extraction ability. After integrating all three modules, the final model achieves 97.6% precision, 96.8% recall, 98.3% mAP_@50_, and 78.5% mAP_@50–95_, while reducing parameters by 28.3% and GFLOPs by 22.5%, and increasing inference speed by 118.8% compared with the baseline. Although precision and recall are slightly lower than those of the original model, the final architecture attains better overall detection and positioning performance under a much lighter and faster configuration. This demonstrates that MobileNetV4 provides the efficiency foundation, C2PSA_MLCA restores discriminative feature representation, and OD_SEConv enhances adaptive convolutional extraction, enabling the proposed model to achieve a favorable balance among accuracy, computational cost, and real-time performance.

#### 3.2.2 Model comparison experiment

**Figure 12:**
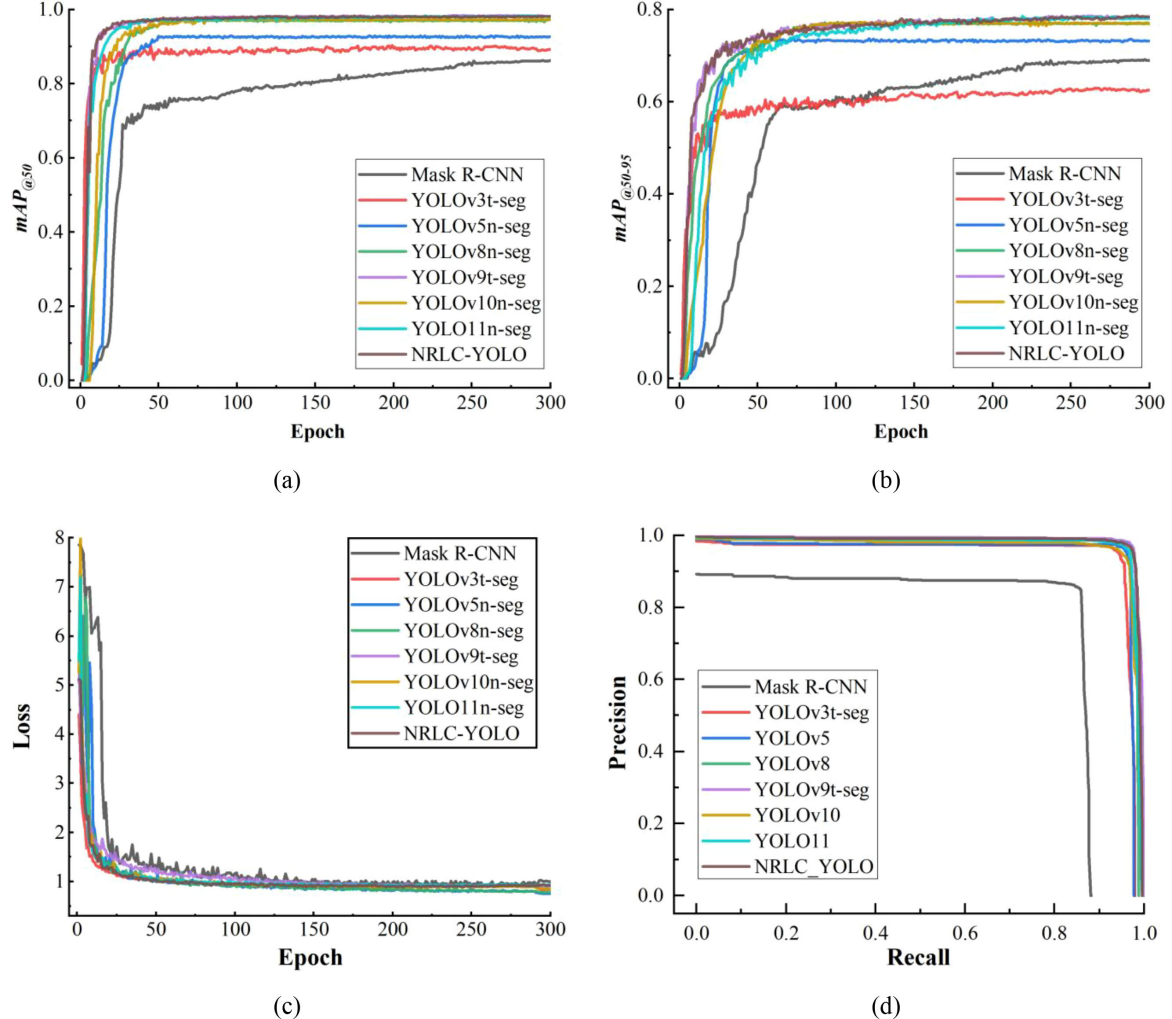
Training results of different models: **(a)** mAP_@50_ curve; **(b)** mAP_@50–95_ curve; **(c)** Loss curve; **(d)** PR curve.

The performance of the designed model is estimated through sufficient contrast experiments with existing typical object detection networks. To ensure a fair and reproducible evaluation, the comparison models in this study were mainly selected from representative off-the-shelf instance-segmentation frameworks. Corresponding experimental data are displayed in **Figure 12**; **Table 4**.

**Table 4:** Experimental results of different models.

Models	P (%)	R (%)	*mAP*_@50_ (%)	*mAP*_@50-95_ (%)	Params (M)	GFLOPs (G)	FPS (f/s)
Mask R-CNN	85.2	87.4	85.1	68.2	25.8	45.1	39.5
YOLOv3t-seg	92.7	88.6	89.2	61.9	10.5	27.9	52.3
YOLOv5n-seg	92.6	93.8	92.6	73.2	5.1	7.8	56.1
YOLOv8n-seg	97.1	96.9	97.3	76.9	6.3	11.4	58.2
YOLOv9t-seg	97.5	97.1	98.2	78.4	22.5	51.7	32.8
YOLOv10n-seg	97.4	96.7	97.2	76.7	7.1	15.2	53.4
YOLO11n-seg	97.7	96.9	98.0	78.2	6.0	10.2	57.9
NRLC-YOLO	97.6	96.8	98.3	78.5	4.4	7.9	126.7

As shown in **Table 4**; **Figure 12**, NRLC-YOLO achieves the best overall performance among the compared models, demonstrating a favorable balance between detection accuracy and efficiency. The proposed model attains the highest mAP@50 of 98.3% and mAP@50–95 of 78.5%, indicating clear advantages in both detection and positioning performance. Compared with Mask R-CNN, mAP@50 and mAP@50–95 are improved by 13.2% and 10.3%, respectively, while compared with YOLOv3t-seg, the corresponding improvements reach 9.1% and 16.6%. Relative to YOLOv5n-seg, the gains remain notable at 5.7% in mAP@50 and 5.3% in mAP@50-95. Compared with the more recent YOLO-based models, NRLC-YOLO still maintains consistent improvements in both metrics. As reflected in **Figures 12a, b**, the proposed model rises rapidly during training and stabilizes at the highest level, which further confirms its superior optimization result and overall detection quality. These results demonstrate that NRLC-YOLO can provide more reliable visual perception for robotic grasping and positioning in complex natural rubber plantation environments.

In terms of precision and recall, NRLC-YOLO reaches 97.6% and 96.8%, respectively, remaining at a high level although these two individual metrics are not the highest among all methods. Compared with YOLO11n-seg, its precision and recall are both 0.1% lower; compared with YOLOv9t-seg, precision decreases by 0.1% and recall by 0.3%; and compared with YOLOv8n-seg, precision is lower by 0.5% and recall by 0.1%. This suggests that the proposed lightweight design introduces a slight reduction in classification confidence and target coverage. However, this decrease is very limited and does not weaken the overall detection performance. On the contrary, the simultaneous improvement in mAP_@50_ and mAP_@50–95_ indicates that NRLC-YOLO achieves more accurate bounding and mask prediction. This trend is also consistent with the loss curve in **Figure 12c**, where NRLC-YOLO shows a smooth downward trajectory and good convergence behavior, indicating stable optimization and effective feature learning throughout the training process.

The PR curve results provide further insight into the performance differences among the models. As shown in **Figure 12d**, YOLOv9t-seg exhibits the most prominent PR curve, indicating its advantage in precision-recall trade-off. Although NRLC-YOLO is slightly inferior to YOLOv9t-seg in this curve, it still remains close to the top-performing group. Considering that NRLC-YOLO achieves higher mAP_@50_ and mAP_@50-95_, the proposed model demonstrates better overall detection and positioning performance. Therefore, despite the slight disadvantage in PR-curve behavior, NRLC-YOLO still outperforms YOLOv9t-seg in terms of comprehensive performance.

#### 3.2.3 Small target detection experiment

To further evaluate the performance of the proposed method on small-scale targets, a dedicated small target detection experiment was conducted. According to the size characteristics in latex cup harvesting scenarios, the targets were divided into three categories: latex drain (extremely small targets), grasp area (very small targets), and non-grasp area (small targets). The detection performance of NRLC-YOLO and the baseline YOLO11n-seg on these categories is summarized in **Table 5**.

**Table 5:** Detection performance on different small-target categories.

Models	Latex drain (extremely small)			Grasp area (very small)			Non-grasp area (small)		
	P (%)	R (%)	AP (%)	P (%)	R (%)	AP (%)	P (%)	R (%)	AP (%)
YOLO11n-seg	96.1	96.6	97.5	98.1	96.8	97.9	98.5	97.1	98.1
NRLC-YOLO	96.4	96.7	97.8	98.1	96.9	98.1	98.2	96.9	98.3

NRLC-YOLO achieves consistent improvements across all small-target categories. For the latex drain, representing the most challenging extremely small target, the AP increases from 97.5% to 97.8%, indicating enhanced detection of weak and fine structures. For the grasp area, categorized as a very small target, the AP improves from 97.9% to 98.1%, suggesting more accurate capture of fine-grained grasp-related regions. For the non-grasp area, corresponding to the small target, NRLC-YOLO also achieves a higher AP of 98.3%, compared with 98.1% for YOLO11n-seg.

From the perspective of precision and recall, NRLC-YOLO maintains a balanced performance across all categories, while achieving slight improvements in either precision or recall depending on the target scale. These results indicate that the proposed model not only improves overall detection accuracy but also enhances robustness in small-target perception.

The observed improvements can be attributed to the joint effect of the introduced modules. In particular, C2PSA_MLCA enhances local–global feature interaction, which is beneficial for capturing weak structural features of extremely small targets. Meanwhile, OD_SEConv improves adaptive feature refinement, allowing the network to better respond to subtle variations in small-scale regions. As a result, NRLC-YOLO achieves more reliable detection performance for small targets under rubber plantation environments.

#### 3.2.4 Grasp center point positioning experiment

**Figure 13:**
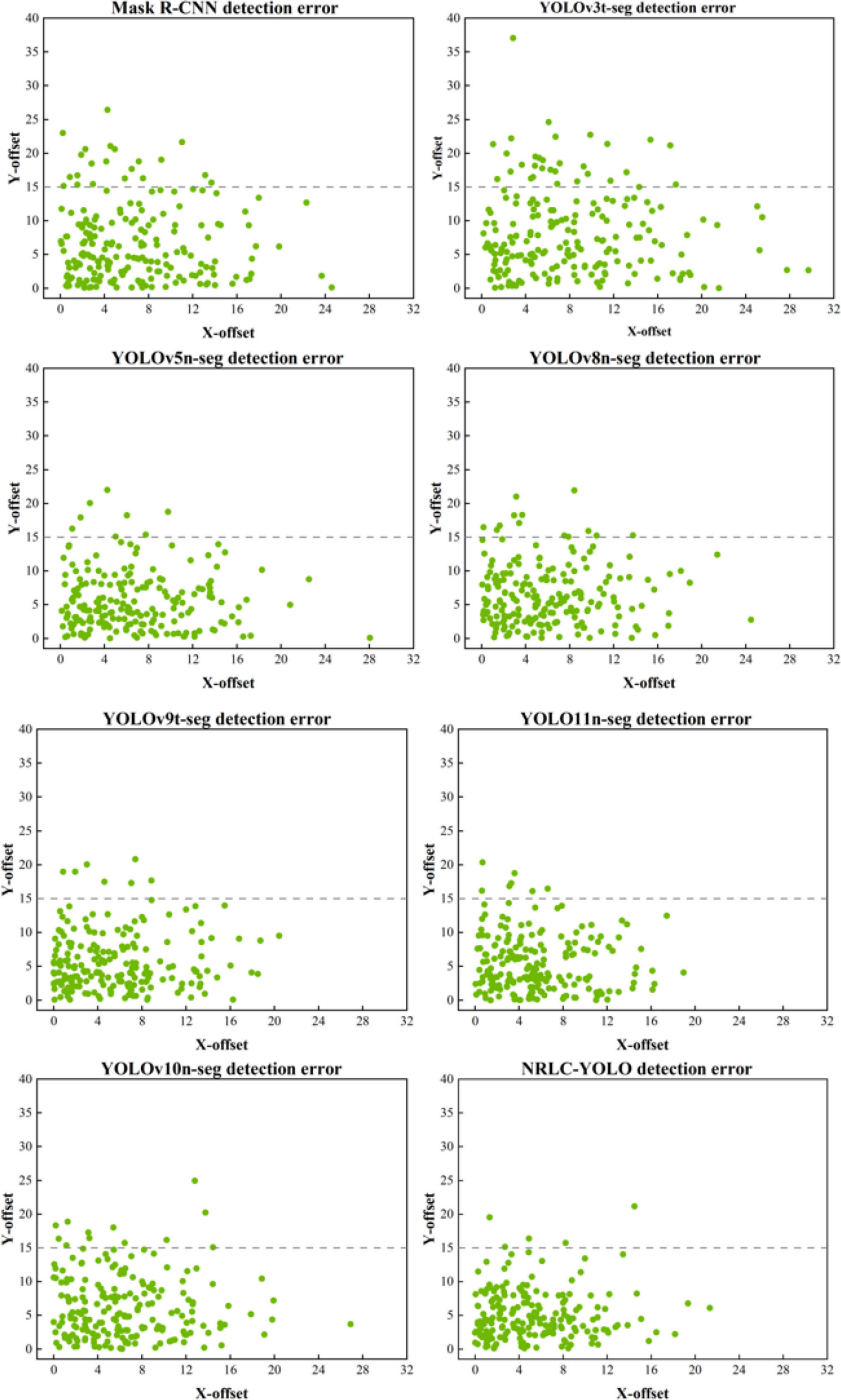
Positioning error distribution of different models.

To validate the effectiveness of the proposed grasp-center positioning strategy, 200 images collected between 8:00 and 10:00 AM were selected for testing. For each model, the predicted grasp-center coordinates were obtained from the corresponding segmentation results, and the positioning error with respect to the ground-truth point was calculated according to Equation 14 in Section 3.2. The prediction errors and results of each comparison model are shown in **Table 6**; **Figure 13**.

**Table 6:** Positioning error statistics of different models.

Model	Mean error (pixel)	Standard deviation	Median error (pixel)
Mask R-CNN	10.63	5.83	9.71
YOLOv3t-seg	12.69	6.65	11.89
YOLOv5n-seg	9.63	5.07	8.89
YOLOv8n-seg	9.84	4.93	9.15
YOLOv9t-seg	8.93	4.90	8.29
YOLOv10n-seg	10.07	5.08	9.87
YOLO11n-seg	8.75	4.41	7.87
NRLC-YOLO	8.08	4.42	7.78

As shown in **Figure 13**, the scatter distributions of the positioning errors exhibit clear differences among models. Mask R-CNN and YOLOv3t-seg show relatively dispersed error points and more large-offset samples, indicating weaker stability in grasp-center localization. In contrast, the lightweight segmentation models generally produce more concentrated error distributions. Among them, YOLO11n-seg and NRLC-YOLO show the most compact scatter patterns, with most samples clustered in the low-error region. In particular, NRLC-YOLO presents fewer high-offset outliers, suggesting that the proposed method can achieve more consistent and robust grasp-center prediction under complex plantation conditions.

As shown in **Table 6**, the proposed NRLC-YOLO achieved the lowest mean positioning error of 8.08 pixels, together with the smallest median error (7.78 pixels) among all compared models. The standard deviation of 4.42 pixels further indicates that the positioning results of NRLC-YOLO remain relatively stable across different test images. In comparison, YOLO11n-seg and YOLOv9t-seg also demonstrated relatively competitive performance, with mean errors of 8.75 pixels and 8.93 pixels, respectively, but both were still slightly inferior to NRLC-YOLO. By contrast, YOLOv5n-seg, YOLOv8n-seg, and YOLOv10n-seg exhibited larger positioning errors, while Mask R-CNN and YOLOv3t-seg showed the weakest performance. These results indicate that the proposed method provides more precise grasp-related region extraction and is therefore more suitable for subsequent center-point determination.

To further evaluate whether the observed reduction in positioning error is statistically reliable, a paired t-test was conducted on the per-image positioning errors using NRLC-YOLO as the reference model. The statistical results are presented in **Table 7**. It can be observed that the positioning error reductions relative to Mask R-CNN, YOLOv3t-seg, YOLOv5n-seg, YOLOv8n-seg, and YOLOv10n-seg are all highly significant (p < 0.001), indicating that NRLC-YOLO achieves a stable and consistent improvement over these models on the same test images. In contrast, the differences relative to YOLO11n-seg (p = 0.114) and YOLOv9t-seg (p = 0.080) do not reach the 0.05 significance level. This can be attributed to the strong baseline performance of these lightweight segmentation models, particularly YOLO11n-seg, which already provides accurate instance segmentation results and competitive positioning accuracy.

**Table 7:** Statistical comparison of positioning errors.

Comparative model	Mean error difference (pixel)	t value	p value	95% CI of difference
YOLO11n-seg	0.67	1.588	0.114	[-0.16, 1.50]
YOLOv9t-seg	0.85	1.761	0.080	[-0.10, 1.80]
YOLOv5n-seg	1.55	3.500	<0.001	[0.68, 2.42]
YOLOv8n-seg	1.76	3.655	<0.001	[0.81, 2.71]
YOLOv10n-seg	1.98	4.266	<0.001	[1.07, 2.90]
Mask R-CNN	2.55	4.929	<0.001	[1.53, 3.56]
YOLOv3t-seg	4.61	7.929	<0.001	[3.46, 5.76]

Nevertheless, NRLC-YOLO still achieves the lowest mean and median positioning errors among all compared models, indicating that the proposed improvements provide additional performance gains on top of an already strong baseline. From a practical perspective, even a small reduction in positioning error can contribute to more stable grasp-center estimation in robotic harvesting tasks. Therefore, the proposed method not only maintains the advantages of the baseline model but also enhances positioning accuracy and robustness under complex plantation conditions.

Furthermore, from the perspective of robotic operation, the positioning accuracy achieved by NRLC-YOLO is sufficient for downstream grasping execution. Since the average error remains within the tolerance range of the robot end-effector, the proposed method can effectively satisfy the practical requirements of latex cup grasp-center positioning and provide reliable support for real-world robotic harvesting applications.

#### 3.2.5 Positioning experiment under complex scenarios

To further evaluate the robustness of the proposed method in practical harvesting conditions, a positioning experiment under complex scenarios was conducted. According to the visual characteristics of latex cups in field environments, the test images were divided into three categories: normal scenario, tilted scenario, partially occluded scenario. For each category, the positioning performance was evaluated in terms of mean error, standard deviation and successful positioning rate. The proposed NRLC-YOLO was compared with the baseline model under the same test protocol.

As shown in **Table 8**, the positioning performance of both models gradually declined as the scene complexity increased from normal to tilted and partially occluded conditions. In the normal scenario, NRLC-YOLO achieved a lower mean positioning error of 8.05 pixels than YOLO11n-seg (8.75 pixels), while the standard deviations of the two models were nearly identical, indicating comparable stability under routine field conditions. In the tilted scenario, the mean errors increased for both models due to pose variation of the latex cups, but NRLC-YOLO still maintained better performance, reducing the mean error from 9.52 to 8.71 pixels and improving the successful positioning rate from 87.4% to 90.5%. In the partially occluded scenario, which was the most challenging case, both models showed the largest errors and lowest positioning rates. Nevertheless, NRLC-YOLO still outperformed YOLO11n-seg, decreasing the mean error from 10.88 to 9.94 pixels and increasing the successful positioning rate from 83.2% to 86.4%. These results demonstrate that the proposed method provides more reliable grasp-center localization, with its advantage becoming more evident under challenging plantation scenarios.

**Table 8:** Grasp center positioning performance under different complex scenarios.

Scenario type	Models	Mean error (pixel)	Standard deviation	Successful positioning rate (%)
Normal	YOLO11n-seg	8.75	4.41	91.3
	NRLC-YOLO	8.05	4.42	93.6
Tilted	YOLO11n-seg	9.52	4.76	87.4
	NRLC-YOLO	8.71	4.53	90.5
Partially occluded	YOLO11n-seg	10.88	5.18	83.2
	NRLC-YOLO	9.94	4.89	86.4

#### 3.2.6 Forest grasping experiment

**Figure 14:**
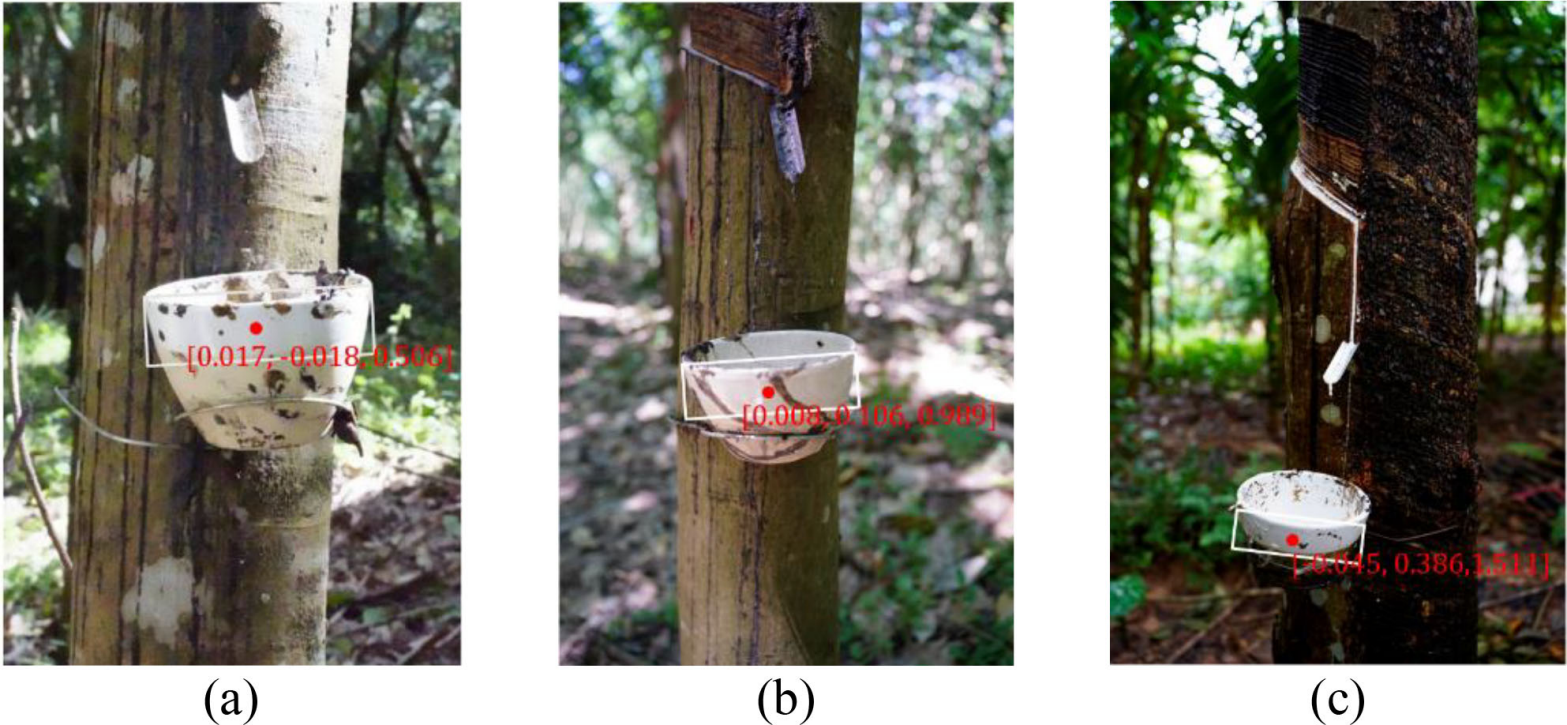
Grasping and detection at different distances: **(a)** 0.5m; **(b)** 1.0m; **(c)** 1.5m.

To evaluate the practical feasibility of the proposed algorithm for latex cup harvesting under field conditions, latex cups from 30 rubber trees were selected for grasping verification experiments. The robotic arm was positioned at three working distances (0.5 m, 1.0 m, and 1.5 m) to assess grasping performance under different operating ranges. The corresponding experimental results are presented in **Figure 14**; **Table 9**. In this study, the grasping success rate is defined as the ratio of successfully executed grasps to the total number of tested targets.

**Table 9:** Grasping test at different distances.

Test distance (m)	Number of successful detections	Number of correct positioning points	Grasping success rate (%)
0.5	30	28	93.3
1.0	28	27	90.0
1.5	25	22	73.3

As shown in **Table 9**, the proposed system achieved the best performance at a distance of 0.5 m, where all 30 latex cups were successfully detected and 28 positioning points were correctly localized, corresponding to a grasping success rate of 93.3%. When the operating distance increased to 1.0 m, the number of successful detections and correct positioning points decreased slightly to 28 and 27, respectively, and the grasping success rate remained at 90.0%, indicating that the method still maintained relatively stable grasping capability within this range. However, at 1.5 m, the number of successful detections dropped to 25, and only 22 positioning points were correctly localized, resulting in a lower grasping success rate of 73.3%. This performance degradation can be attributed mainly to the reduced depth measurement accuracy of the RGB-D camera for distant targets, together with the increased influence of viewpoint deviation and end-effector reach limitations, which jointly reduced the reliability of positioning matching and final grasp execution. Overall, the results demonstrate that the proposed method provides reliable latex cup detection, localization, and grasping performance at short to medium operating distances, especially within 1.0 m, and therefore satisfies the practical requirements of robotic harvesting in forest environments.

## 4 Discussion

### 4.1 Visual analysis

The performance improvement of NRLC-YOLO is not simply the result of combining several lightweight modules, but can be explained by the complementary roles of the introduced components. In latex cup harvesting, the perception model must simultaneously satisfy three requirements: lightweight deployment on robotic platforms, sufficient discrimination of small target features, and robustness to complex plantation backgrounds. From this perspective, MobileNetV4 mainly contributes to reducing model parameters and computational burden, thereby improving deployment efficiency. However, lightweight backbone replacement may also weaken the preservation of fine-grained features, especially for small latex cup targets. To compensate for this limitation, C2PSA_MLCA is introduced to enhance feature representation through the joint modelling of local detail information and global contextual dependencies, which helps the network focus more effectively on key target regions. On this basis, OD_SEConv further improves adaptive feature refinement by combining channel recalibration and dynamic convolution, thereby enhancing robustness to target variation, partial occlusion, and background clutter. Therefore, the three modules form a progressive mechanism of lightweight compression, representation compensation, and adaptive refinement, which explains the overall improvement of NRLC-YOLO over the baseline model.

**Figure 15:**
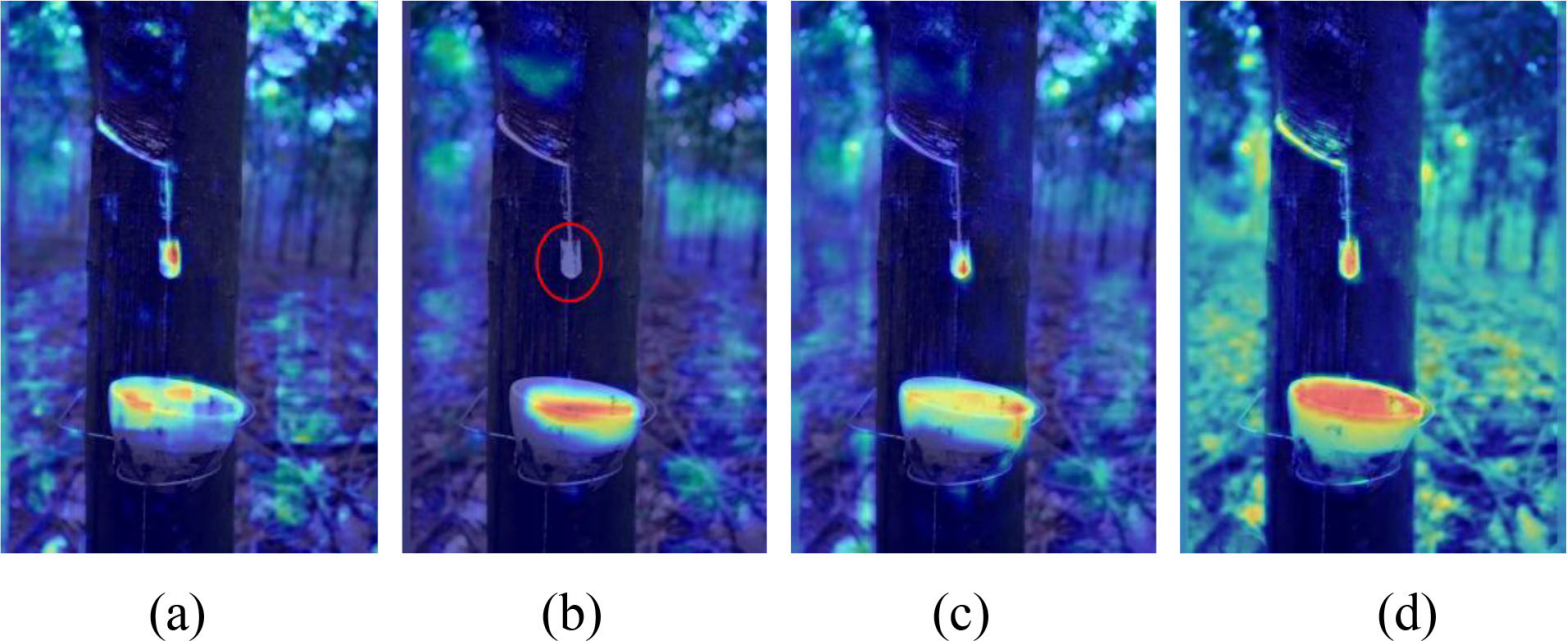
Model heatmap: **(a)** Baseline; **(b)** MobileNetV4; **(c)** MobileNetV4+C2PSA_MLCA; **(d)** MobileNetV4+C2PSA_MLCA+OD_SEConv.

To visually illustrate the feature extraction behavior of different model variants, heatmaps were introduced during the detection process, as shown in **Figure 15**. The heatmaps clearly reflect the response intensity of the models to latex cup and latex drain regions. After replacing the original backbone with MobileNetV4, the response to small target regions becomes weaker, and some latex drain areas show insufficient activation, indicating that lightweighting alone may reduce fine-grained feature representation. After introducing C2PSA_MLCA, the attention to latex cup and latex drain regions becomes more concentrated, suggesting that the module effectively compensates for the representation loss caused by backbone lightweighting. With the further incorporation of OD_SEConv, the activation over the target regions becomes clearer and more stable, while irrelevant background responses are suppressed to a certain extent. These observations are consistent with the ablation results, indicating that the proposed modules improve the model not only in quantitative metrics but also in feature discrimination capability. Therefore, the visual analysis further verifies the rationality of the proposed NRLC-YOLO design for latex cup detection under complex plantation conditions.

### 4.2 Comparative analysis under different lighting conditions

**Figure 16:**
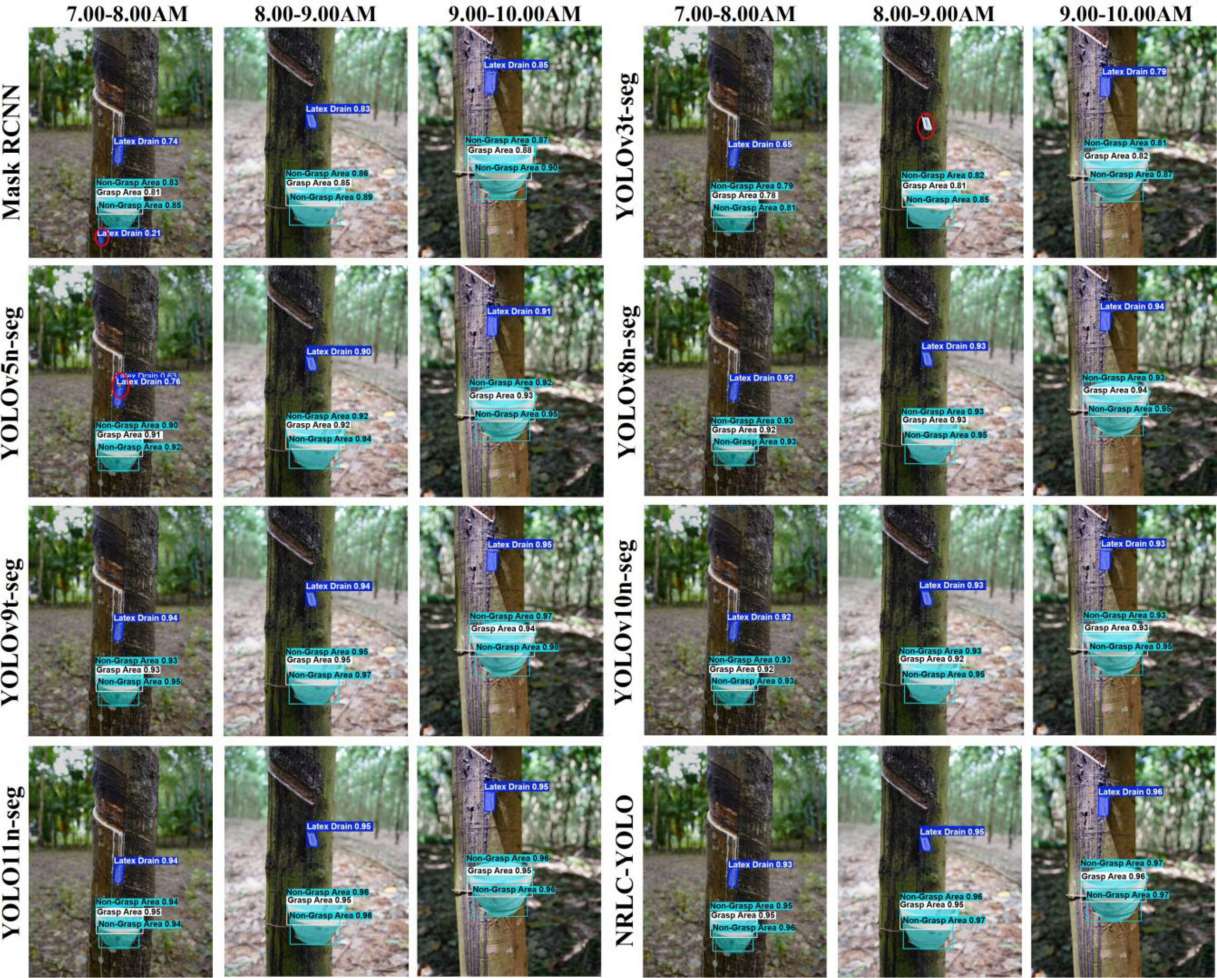
Model comparison experiments under different lighting conditions.

Considering the lighting conditions during the robot’s latex cup grasping operation, we selected images collected at three different time periods from the dataset and tested different models. The results are shown in **Figure 16**. The experimental results show that detection performance is best between 9:00 and 10:00 AM, when there is ample lighting. However, between 7:00 and 8:00 AM, due to the sun’s recent rise and obstruction by the leaves of the rubber plantation, the light is generally insufficient, resulting in missed and false detections of small objects, such as those achieved by Mask R-CNN, YOLOv3t-seg and YOLOv5n-seg. Furthermore, the detection precision of the latex cup position between 8:00 and 9:00 AM is comparable to that between 9:00 and 10:00 AM, indicating that the lighting conditions between 8:00 and 10:00 AM are suitable for the latex cup grasping task.

### 4.3 Comparative analysis under different rubber plantation scenarios

**Figure 17:**
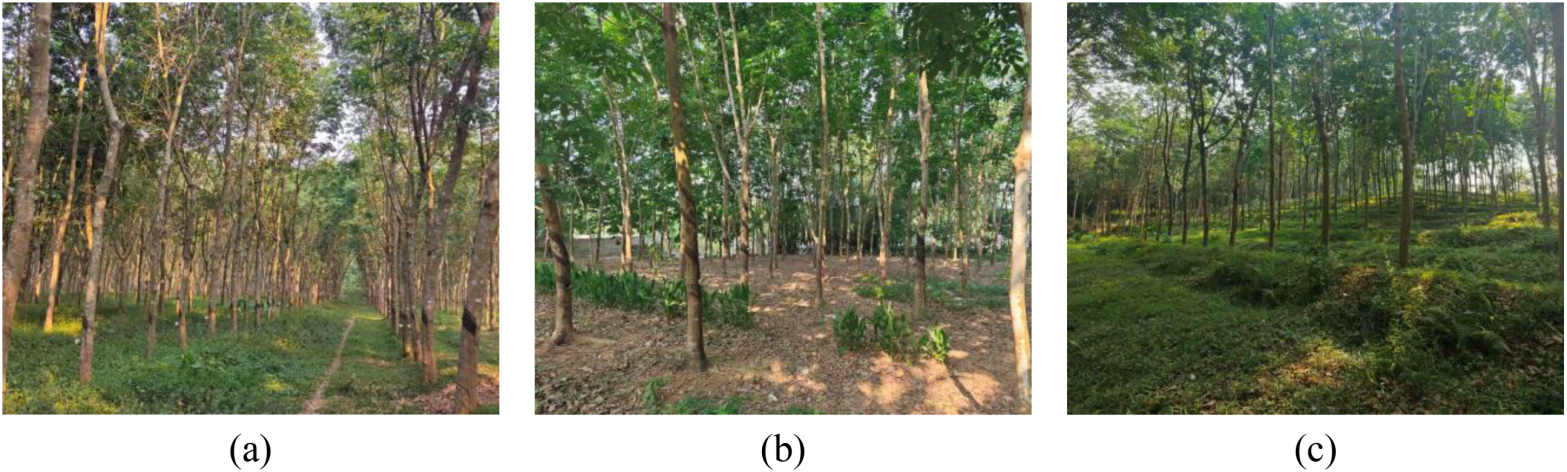
Three rubber plantation scenarios: **(a)** Standardized plantation; **(b)** Non-standard plantation; **(c)** Sloped plantation.

To further examine the robustness of the proposed method under practical field conditions, three representative rubber plantation scenarios were considered, namely a standardized plantation, a non-standard plantation, and a sloped plantation, as illustrated in **Figure 17**. These scenarios reflect typical differences in planting regularity and terrain conditions encountered during field harvesting.

The quantitative results in **Table 10** show that the proposed method maintains highly stable perception performance across the three plantation scenarios. The detection time remains almost unchanged at approximately 0.0175 s, while the mAP_@50_ fluctuates only slightly, within a narrow range around 98%. This consistency suggests that the visual perception module is not strongly affected by differences in plantation layout. In other words, the proposed NRLC-YOLO exhibits good robustness to variations in rubber plantation structure, including changes from standardized planting conditions to more irregular field arrangements.

**Table 10:** Detection efficiency and task success across plantation scenarios.

Scenario	Detection time (s)	*mAP*_@50_ (%)	Successful positioning rate (%)
Standardized plantation	0.0175	98.3	93.6
Non-standard plantation	0.0174	98.1	91.7
Sloped plantation	0.0175	98.3	92.9

By contrast, the successful positioning rate shows a slightly larger variation than the detection metrics, decreasing from 93.6% in the standardized plantation to 91.7% in the non-standard plantation and 92.9% in the sloped plantation. Although the overall fluctuation remains limited, this trend indicates that changes in field conditions influence the final task execution more than the detection stage itself. A plausible explanation is that plantation irregularity and terrain variation mainly affect the spatial consistency of the robotic positioning process, rather than the visual recognition capability of the model.

In particular, the sloped plantation deserves further attention. While its detection time and mAP_@50_ remain comparable to those of the other scenarios, terrain variation may introduce additional challenges to the robotic system, such as viewpoint instability, changes in relative target geometry, and reduced motion stability during grasp execution. Therefore, the present results suggest that the proposed method is sufficiently robust at the perception level across different plantation scenarios, whereas future improvements should place greater emphasis on system-level adaptation to terrain complexity, especially for robotic operation in sloped environments.

Overall, the results indicate that differences among rubber plantation scenarios have only a limited effect on detection performance, whereas the influence of terrain is more likely to emerge during the physical interaction and execution stages of the harvesting process. This distinction is important for future system optimization, as it suggests that further performance gains may depend less on perception accuracy itself and more on improving the adaptability of the robotic platform under complex field conditions.

### 4.4 System energy consumption and cost analysis

In addition to precision, energy consumption and deployment cost are also important for practical robotic harvesting in plantation environments. Since latex cup harvesting is performed in large-scale outdoor fields, the perception model should operate efficiently on resource-constrained platforms.

**Figure 18:**
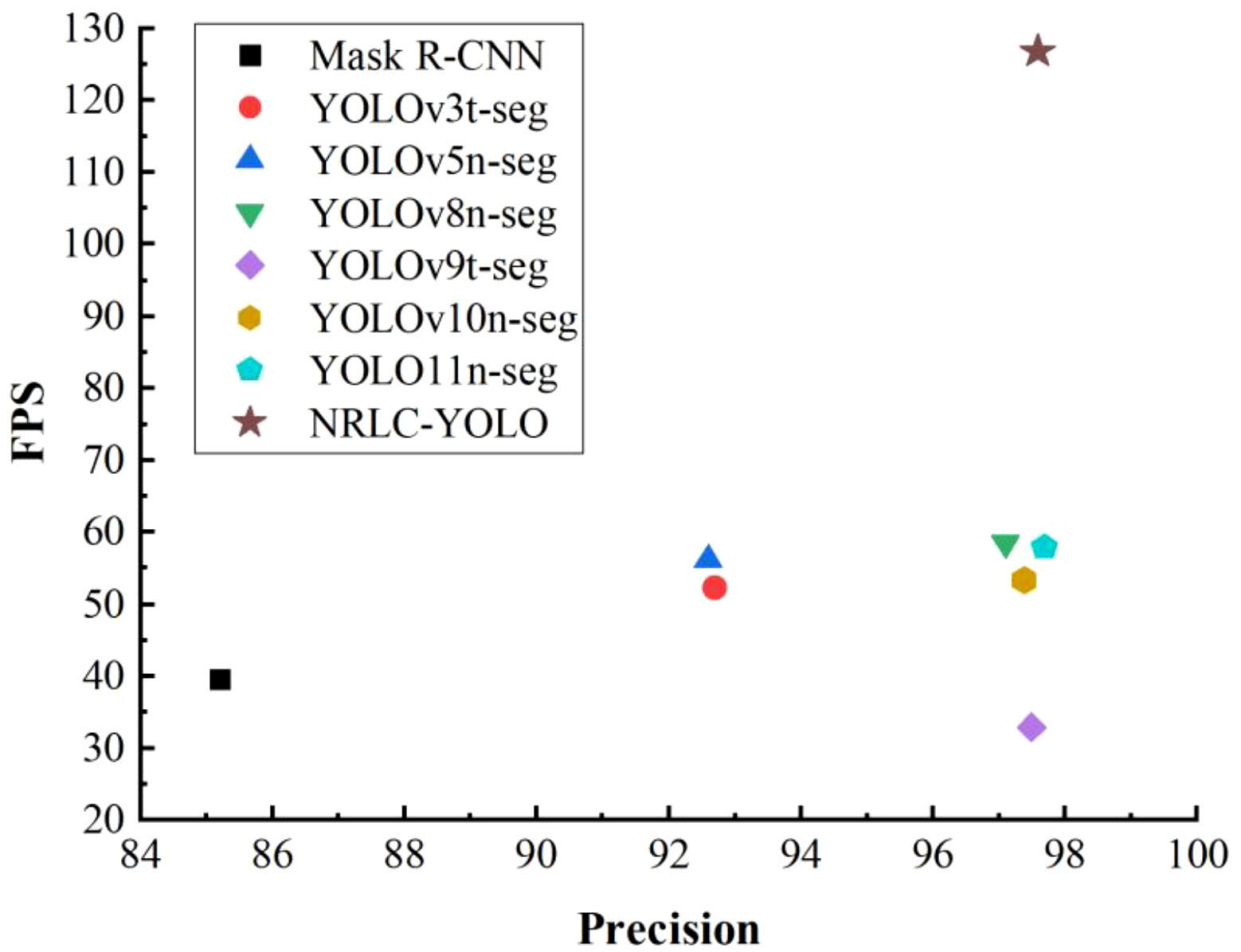
Precision-FPS curve.

NRLC-YOLO shows clear advantages in lightweight design and real-time performance. The proposed model contains only 4.4 M parameters and requires 7.9 GFLOPs, which are lower than those of most compared methods. It also achieves 126.7 FPS, exceeding YOLO11n-seg, YOLOv8n-seg, and YOLOv10n-seg by 68.8 FPS, 68.5 FPS, and 73.3 FPS, respectively. This allows the system to complete inference with lower computational burden and shorter active processing time, which is beneficial for reducing energy consumption during continuous operation. As shown in the Precision-FPS trade-off curve (**Figure 18**), NRLC-YOLO maintains high precision while delivering much higher inference speed. In addition, the proposed perception-driven framework combines deep learning-based target perception with lightweight geometric localization, which helps reduce hardware requirements and system complexity. Therefore, the proposed method provides a favorable balance between accuracy, efficiency, and deployment cost, making it more suitable for robotic latex cup harvesting in field environments.

## 5 Conclusions

This study proposed NRLC-YOLO for detecting latex cups and localizing grasp-related regions in rubber plantation environments. By combining instance segmentation with explicit grasp-center localization, the proposed framework provides reliable visual support for automated latex cup grasping and harvesting. The main conclusions are as follows:

1. NRLC-YOLO was developed by introducing MobileNetV4, C2PSA_MLCA, and OD_SEConv into the YOLO11-seg framework. Experimental results show that the proposed model reduces the parameter count to 4.4 M while achieving an inference speed of 126.7 FPS, demonstrating clear advantages in lightweight design and real-time deployment capability for robotic platforms.
2. A lightweight grasp center positioning strategy was designed based on the segmented grasp-related regions. The experimental results show that the proposed method achieves an average positioning error of 8.08 pixels, indicating good positioning accuracy for robotic grasping. Field experiments further verified the practical feasibility of the proposed framework, with a grasping success rate of up to 93.3% under real plantation conditions.
3. Comparative experiments under different illumination conditions, small-target categories, and complex scenarios demonstrate that NRLC-YOLO maintains robust detection and positioning performance in realistic rubber plantation environments. In particular, the proposed model shows better adaptability in the detection of small targets such as latex drains and achieves more reliable grasp center positioning under tilted and partially occluded scenarios. These results confirm its practical value for improving the automation level of latex cup harvesting.

Although encouraging results were achieved in this study, several limitations still remain. The current dataset does not include sufficient weather-diverse samples, and severely occluded cases are relatively limited. In addition, the overall harvesting process from perception to execution still requires further improvement in efficiency. Future work will therefore focus on cross-weather validation, more challenging occlusion scenarios, and further optimization of the latex cup harvesting robot for faster and more reliable field operation.

## Data Availability

The original contributions presented in the study are included in the article/supplementary material. Further inquiries can be directed to the corresponding author.
